# Genotoxic, Histopathological and Oxidative Stress Responses in Catfish, Clarias gariepinus, Exposed to Two Antifouling Paints

**DOI:** 10.5696/2156-9614-7.16.71

**Published:** 2017-12-18

**Authors:** Ochuwa O. George, Nnamdi H. Amaeze, Emmanuel Babatunde, Adebayo A. Otitoloju

**Affiliations:** 1 Environmental Toxicology and Pollution Management Laboratory, Department of Zoology, University of Lagos, Nigeria; 2 Fisheries Unit, Department of Marine Sciences, University of Lagos, Akoka-Yaba, Nigeria

**Keywords:** biocides, non-target organisms, maritime industry, aquatic pollution, biomarker, histopathology

## Abstract

**Background.:**

Antifouling paints are enriched with biocides and employed in the maritime industry to protect moving and fixed surfaces from fouling activities of sea dwelling invertebrates. There is limited information on their effect on the non-target African catfish, Clarias gariepinus, a commonly consumed fish in Lagos.

**Objectives.:**

This study investigated the effects of two commonly used antifouling paints (Berger TBT-free (A/F783 (H)), reddish brown color and Silka Marine lead based paint, pale orange color) on a non-target catfish species, Clarias gariepinus.

**Methods.:**

The study involved an initial 96-hour acute toxicity assay followed by chronic toxicity evaluation (using 1/10th and 1/100th 96-hour median lethal concentration (LC_50_) values) for 28 days to determine the ability of the paints to induce micronucleus and red blood cell abnormalities, and histopathological as well as oxidative stress effects in the catfish.Examined anti-oxidative stress enzyme activities include superoxide dismutase (SOD), catalase (CAT), reduced glutathione (GSH) and glutathione-s-transferase (GST).

**Results.:**

Acute toxicity evaluation results indicated that the Berger paint was 16.1-times more toxic than Silka paint with 96-hour LC_50_ values of 0.71 mg/L and 11.49 mg/L, respectively. Results from the biochemical assay indicated significantly higher (P<0.05) levels of a lipid peroxidation product, malondialdehyde, in Silka-exposed catfish compared to the control. All enzymes showed significantly higher activities in Berger paint-exposed catfish compared to the control. There was evidence of micronucleated and binucleated cells in the red blood cells of fish exposed to both paints. Histopathological assessment indicated that the exposed fish gills showed evidence of abnormalities such as curved lamellae epithelial necrosis, epithelial lifting and hyperplasia. The liver samples of the catfish showed evidence of portal inflammation as well as mild to severe steatosis, while the gonads showed varying percentages of follicle degeneration.

**Conclusions.:**

The present study combined an array of biomarkers to determine the negative health impacts of two commonly used antifouling paints on non-target catfish inhabiting Lagos Lagoon. Further in situ studies are recommended to determine the current status of the lagoon fish.

**Ethics Approval.:**

Ethical approval was obtained from the Department of Zoology, University of Lagos, Post-Graduate Committee. Note that this work commenced before the establishment of the University of Lagos Ethical Committee for the use of animals and humans in scientific studies. The committee does not give retroactive approval but stands by existing approvals before its establishment. However, this study followed the World Medical Association principles on the treatment of animals used in research (https://www.wma.net/policies-post/wma-statement-on-animal-use-in-biomedical-research/), and also American Fisheries Society Guidelines for the Use of Fishes in Research (https://fisheries.org/policy-media/science-guidelines/guidelines-for-the-use-of-fishes-in-research/)

## Introduction

Organisms such as mussels, barnacles, bryozoans and algae are referred to as biofouling organisms and are a source of concern for boat owners.^1^ They attach to the hull of ships, causing increased friction, leading to lower speed, impaired maneuverability and increased fuel consumption, resulting in economic loss.^2^,^3^ The competition for living space in the marine environment is intense, and all surfaces, static or mobile, are susceptible to fouling. The colonization of living or non-living surfaces by sessile organisms, plants or animals is an omnipresent phenomenon in aquatic environments.^4^

Over the years, antifouling paints have been introduced as a means of combating biofouling organisms. As a result, antifouling paints have become important sources of biologically active heavy metal pollution in the aquatic ecosystem. These paints are used in aquaculture and shipping industries to prevent bio-fouling. Some examples of biocides present in antifouling paint include lead (Pb), copper (Cu), zinc (Zn) and organic tin compounds (organotin) such as tri-butyl-tin (TBT) and tri-phenyl-tin (TPT).^5^ Concerns regarding the effects of TBT on non-target aquatic organisms resulted in its worldwide ban in antifouling paints for most ships from January 2003 based on the International Maritime Association Assembly Resolution A.895 (21) of November 1999.^6^ To this end, TBT-free antifouling paints have gained popularity and are now the only legal types of antifouling paints permissible for vessels in many countries. Currently, due to numerous bans and legal sanctions, a decrease in the inflow of additional organotin compounds to sediments has been recorded. However, there are areas that remain exposed to this form of pollution, such as shipyards, harbors and passenger ship terminals which constitute a potential pollution source for the marine environment due to intensive shipping, reception of various wastes from ships, cargo loading and unloading operations, and ship building and repair/remodelling.^7^ In developing countries like Nigeria, illegal use of TBT-based antifouling paint is reportedly practiced by local boat merchants (based on personal interactions) and regulation appears to be ineffective.

Many novel antifouling paints remain heavy metal-based and may have negative effects on non-target species. Espinet al.^8^ noted the potency of heavy metals in the formation of reactive oxygen species (ROS) capable of inducing oxidative damage to lipids, DNA and proteins. In the present study, two commonly used antifouling paints (Berger and Silka) in Nigeria were evaluated for their genotoxic, histopathologic and oxidative stress potential to determine their effects on African catfish, Clarias gariepinus, which is widely harvested in the Lagos Lagoon for human consumption. Katranitsas et al.^9^ reported decreased enzymatic activities in brine shrimp exposed to polyvinylchloride (PVC) vessel structures painted with Flexguard VI-II, an indication that toxicants in the antifouling paint matrixes can leach into the surrounding waters, intoxicating non-target fauna. Commonly employed oxidative stress biomarkers in aquatic ecosystem monitoring include glutathione-s-transferases, superoxide dismutase, catalase, reduced glutathione and the lipid peroxidation biomarker malondialdehyde.^10^,^11^ These biomarkers reflect the biochemical status of organismal systems with respect to countering the onslaught of free radicals and reactive oxygen species, of which many are heavy metals.^12^ Oxidative stress results in cellular damage often associated with tissue lesion and structural damage to organs.^13^ Reports abound on the histopathological effects of heavy metal exposures to fish tissues.^14^ Together with chromosomal damage, histopathology reflects far reaching effects on biological systems which are associated with metal-induced oxidative stress.^15^

**Abbreviations**
*CAT*: Catalase
*GSH*: Glutathione
*GST*: Glutathione-s-transferase
*LC*_*50*_: Median lethal concentration
*MDA*: Malondialdehyde
*ROS*: Reactive oxygen species
*SOD*: Superoxide dismutase
*TBT*: Tributyltin

Fish is a staple food in coastal cities like Lagos.^16^ Any impairment of fish health could reduce yield and contribute to food shortages.

The present study combines a battery of such biomarkers to determine possible negative health impacts of two commonly used antifouling paints on the catfish inhabiting Lagos Lagoon. Catfish are part of the fish stock of the Lagos Lagoon, a water body which is notable for fishing activities, among other uses.^16^ The nutritional value of this fish species was emphasized by Famurewa et al.,^17^ who indicated that catfish contain 40.53 to 70.00% protein.

## Methods

Juvenile catfish were exposed to two antifouling paints available in Lagos markets, Berger and Silka, over 4 days and 28 days to determine their acute and chronic toxicity effects, respectively, on non-target species inhabiting the Lagos Lagoon. A control experiment also involving juvenile catfish (from the same batch as the exposed) was simultaneously maintained in dechlorinated tap water for the same periods and under the same laboratory conditions. After exposure to high concentrations during these acute toxicity studies, 96-hour median lethal dose (LC_50_) was determined using probit analysis and fractions of the 96-hour LC_50_ were exposed to fish during the chronic toxicity test to determine possible sub lethal effects of exposure. The 96-hour LC_50_ is the concentration which results in mortality of 50% of the catfish after 96 hours of exposure.

### Test Organisms

Juvenile Clarias gariepinus, 8 weeks old, (length 23.0 ± 1.0 cm, total weight 45.0 ±2.0 g) were procured from a fish farm in Lagos and transported to the Ecotoxicology Laboratory, Department of Zoology, University of Lagos in plastic bags half full with pond water. The fish were acclimatized to laboratory conditions (temperature 28 ± 2°C and relative humidity 70 ± 2%) in 50 L plastic tanks.

### Preparation of Test Compounds

The test compounds were Silka marine Pb-based paint and Berger TBT-free A/F783 (H) Cu/Zn/Pb-based antifouling paints. These brands were selected for this study because an informal survey indicated they are the two most commonly used paints. They were each introduced into the test medium using syringe tubes of different sizes (1 mL, 2 mL, 5 mL and 20 mL). Each desired working concentration was prepared by adding the paints to the bioassay tank and mixing vigorously to form each test media.

### Acute toxicity testing

Initial range-finding tests of the antifouling paint on C. gariepinus indicated that 0% and 100% mortality occurred between 5 and 17 mg/L and 0.25 and 5.0 mg/L solutions of Silka and Berger paint, respectively. Thus, six Silka paint concentrations (5.0, 7.0, 10.0, 12.0, 15.0, and 17.0 mg/L) and Berger paint concentrations (0.5, 0.6, 0.7, 0.8, 0.9 and 1.0 mg/L) were selected for further testing. A total of 10 juvenile fish were distributed in each test concentration and control respectively, with care taken to minimize animal handling. Percentage mortality was recorded at intervals of 24-, 48-, 72- and 96-hours. The bioassay was conducted using transparent glass tanks (height 19 cm; width 15 cm).

### Chronic Toxicity Testing of Antifouling Paints

Two sub-lethal concentrations of 96-hour LC_50_ (11.490 mg/L) of Silka antifouling paint determined by probit analysis, 1.149 mg/L (1/10th 96-hour LC_50_) and 0.1149 mg/L (1/100th 96-hour LC_50_) were selected for the chronic exposure. Two sub-lethal concentrations of 96-hour LC_50_ of Berger paint were determined at 0.71 mg/L (1/10th 96-hour LC_50_) and 0.0071 mg/L (1/100th 96-hour LC_50_). Both concentrations were each exposed to juvenile catfish for 28 days to determine their sub lethal effects on the non-target species in the glass bioassay tanks. The experiment was conducted following the Organization for Economic Cooperation and Development (OECD) Guidelines for Testing of Chemicals, Fish Acute Toxicity, no. 203.^18^ After the 28-day exposure period, three specimens per concentration were analyzed for various sub-lethal effects.

### Micronucleus Assay

Peripheral blood samples were collected by syringe from the caudal vein of the experimental fish. Two slides were prepared per fish/concentrations and they were processed following the steps outlined by Jiraungkoorskul et al.^19^ Briefly, the smeared slides were allowed to air-dry overnight at room temperature, later fixed in absolute ethanol and allowed to dry for 15 minutes. They were then stained with May-Grunwald stain for 10 minutes, then counter stained with 5% Giemsa for another 10 minutes, rinsed very slightly through running tap water and then allowed to dry. The slides were analyzed at 100× (oil immersion) for micronucleus and other nuclear abnormalities under a light microscope (Olympus CHC Model). The micronuclei were characterized by the presence of a small cell inclusion detached from a larger definite nucleus, while those with two joined nuclei of equal size were defined as binucleated. The cells were counted using a hand-held counter (Counter Compass: No. 7777, China).

### Histological Studies

After the 28-day bioassay exposure period, the catfish were dissected to obtain the gills, liver and gonads of the exposed and control individuals. The organs were stored in Boiun's fluid prior to assessment. They were later washed with 70% ethanol and dehydrated through a graded series of ethanol. The organs were serially sectioned at 7 μm in a rotary microtome according to the technique used by Egonmwan.^20^ The fixed section was stained using hematoxylin and eosin and viewed under light microscope. The number of abnormalities detected per section were counted in relation to the total number of cells observed and the values were represented as percentages.

### Measurement of Oxidative Stress Markers

The liver samples of exposed and control catfish were also assessed for oxidative stress markers- superoxide dismutase (SOD), catalase (CAT), glutathione (GSH), Glutathione-s-transferase (GST) and the lipid peroxidation product, malondialdehyde (MDA).

Super oxide dismutase was determined by its ability to inhibit the auto-oxidation of epinephrine, determined by the increase in absorbance at 480 nm as described by Sun and Zigma.^21^ Catalase activity was determined by adopting the methods of Aksenes and Njaa.^22^ The GSH content of non-protein sulfhydryl was estimated according to the method described by Sedlak and Lindsay.^23^ The method of Habiget al.,^24^ was used to measure GST activity, while MDA, an index of lipid peroxidation, was determined by thiobarbituric acid reaction (TBARS) assay using the method of Buege and Aust.^25^

### Statistical Analysis

Results obtained from the acute toxicity assay were subjected to probit analysis using SPSS version 21 (SPSS Inc, Chicago, Illinois) to determine the 96-hour LC_5_, LC_50_ and LC_95_ for both paints. Levels of significance of compared variables from the biochemical and micronucleus assays were determined by one-way analysis of variance (ANOVA) (at P<0.05) and significant means were separated by least significant difference and Duncan's new multiple range test.

### Ethics Approval

Approval was obtained from the Department of Zoology, University of Lagos, Post-Graduate Committee. Note that this work commenced before the establishment of the University of Lagos Ethical Committee for the use of animals and humans in scientific studies. The committee does not give retroactive approval but stands by existing approvals before its establishment. However, this study followed the World Medical Association principles on the treatment of animals used in research (https://www.wma.net/policies-post/wma-statement-on-animal-use-in-biomedical-research/), and also American Fisheries Society Guidelines for the Use of Fishes in Research (https://fisheries.org/policy-media/science-guidelines/guidelines-for-the-use-of-fishes-in-research/)

## Results

The result for the acute toxicity assessment of two commonly available antifouling paints in the Lagos market indicated that Berger antifouling paint with a 96-hour LC_50_ value of 0.710 mg/L was 16.18-times more toxic to the catfish than Silka antifouling paint (96-hour LC_50_, 11.49 mg/L). The toxicity of the paints decreased with duration of exposure over the 96-hour study period and mortality was dose dependent.

**Table 1 —:**
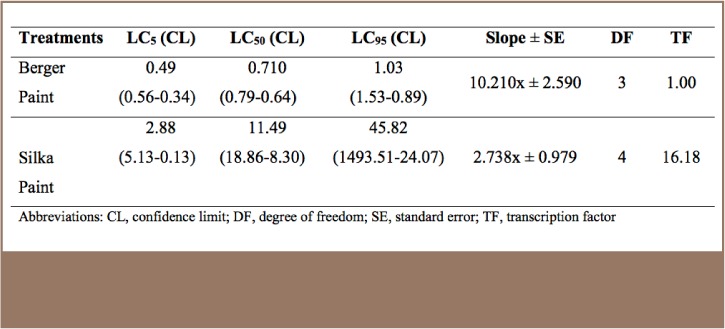
Relative 96-hour Acute Toxicity of Berger and Silka Antifouling Paint on Catfish, Clarias gariepinus (mg/L)

The results obtained indicated that the number of micronucleated cells, immature cells (reticulocytes) and binucleated cells in the controls (A, B and C) were within the ranges observed in untreated animals in studies of this nature (*Table 2*).

**Table 2 —:**
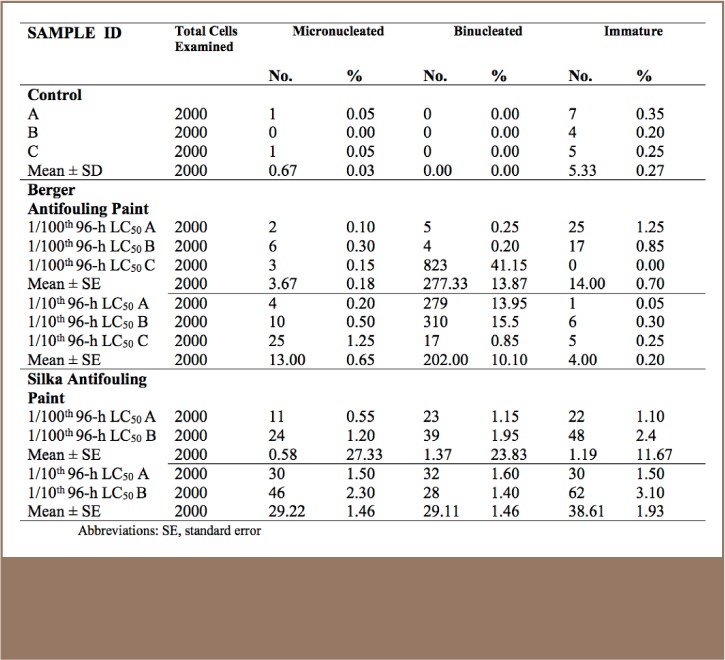
Micronucleus and other red blood cells abnormalities induced in the blood of catfish, C. gariepinus, exposed to sub lethal concentrations of Berger and Silka antifouling paint for 28 days

### 

#### Berger Antifouling Paint

The results of the piscine micronucleus assay in catfish exposed to the Berger antifouling paints indicated different levels of anomalies including micronucleated, binucleated and lysed cells. There were unusually high numbers of binucleated cells in the red blood cells of some catfish exposed to the 1/100th and 1/10th 96-hour LC_50_ of Berger antifouling paints. Also notable were unusually sticky red blood cells, clumped together, in those fish exposed to 1/10th 96-hour LC_50_ concentrations of Berger paint. There were also a number of cells whose membranes appeared damaged among the 1/10th 96-hour LC_50_ Berger paint-exposed group.

#### Silka Antifouling Paint

The results of the micronucleus assessment of Silka antifouling paint-exposed catfish were indicative of the high mutagenic potential of this paint because most of the exposed catfish had evidence of micronucleus induction and binucleated cells. Micronucleus induction tends to be dose dependent, given that the catfish exposed to higher concentrations (1/10th 96-hour LC_50_) equally had higher numbers of micronucleated cells. With respect to micronucleus induction, there was a significant difference between the exposed catfish and control group (*Table 2*).

**Figure 1 —:**
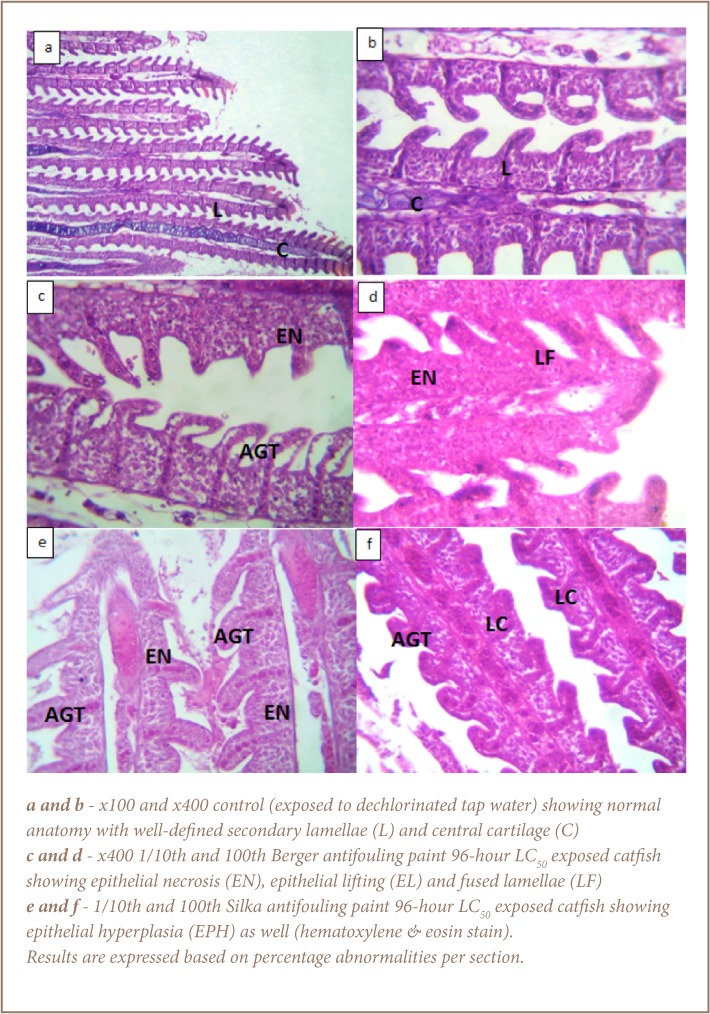
Gill histology of Clarias gariepinus after 28 day study

**Figure 2 —:**
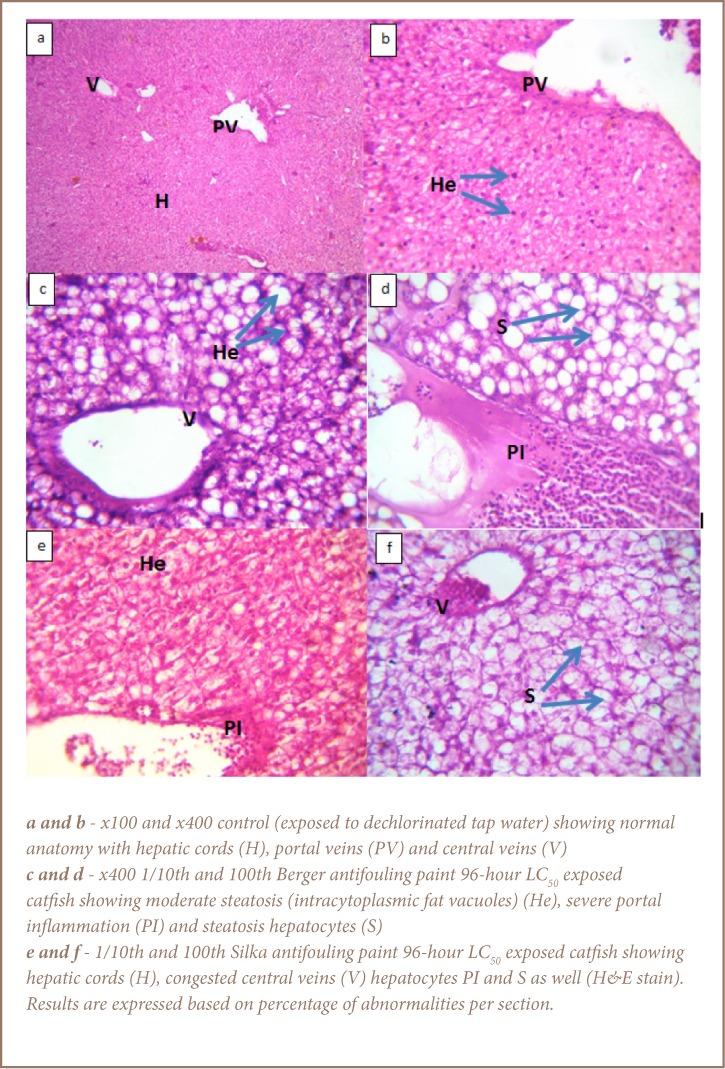
Liver histology of Clarias gariepinus after 28-day study

**Figure 3 —:**
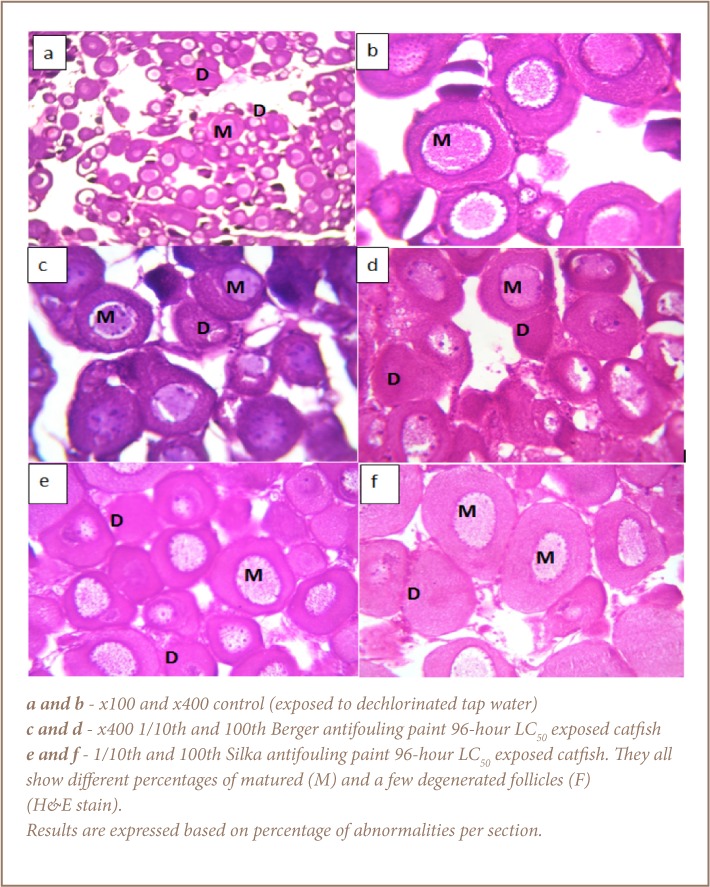
Gonad histology of Clarias gariepinus after 28-day study

Histological assessments indicated that the control catfish had normal anatomy of the gills, liver and gonads (*Figures 1–3*).

The results of the histopathological assessments of catfish exposed to the Berger antifouling paint is indicative of moderate to severe impacts to the gill and liver tissues, but a generally mild effect on the ovaries (*Figures 1–3*). The abnormalities observed in the gills of the Berger paint-exposed catfish include fused lamellae, curved lamellae, necrosis, epithelial lifting and epithelial necrosis (*Figure 1*). In the liver samples of the exposed catfish, there was evidence of structural damage, including hepatocytes with moderate to severe steatosis, congestion of the central veins and portal inflammation (Figure 2). The gonads in this group appeared relatively undamaged with evidence of matured follicles and few degenerated follicles as well (*Figure 3*). Specifically, 20% of the gonads observed in both Berger paint concentration exposed groups (1/10th and 1/100th 96-hour LC_50_) showed evidence of degenerated follicles.

With respect to the catfish exposed to Silka paint, there was also evidence of damage to the gill architecture, which included curved secondary lamellae, lamellae collapse, epithelial lifting, necrosis and hyperplasia (*Figure 1*). The liver samples of the catfish exposed to Silka paint were also impacted. Observed effects included congestion of the central veins, moderate to severe hepatocyte steatosis and portal inflammation (*Figure 2*). The damage in the liver samples was particularly dose dependent, with evidence of severe steatosis on 95% of the cell surface. Only 10% of the observed gonad sections showed degenerated follicles for those fish exposed to 1/100th 96-hour LC_50_ concentration of Silka paint, while 40% of those exposed to 1/10th 96-hour LC_50_ of Silka paint showed evidence of degenerated follicles.

### Oxidative Stress Responses in Gill and Liver Samples of Exposed Catfish

**Figure 4 —:**
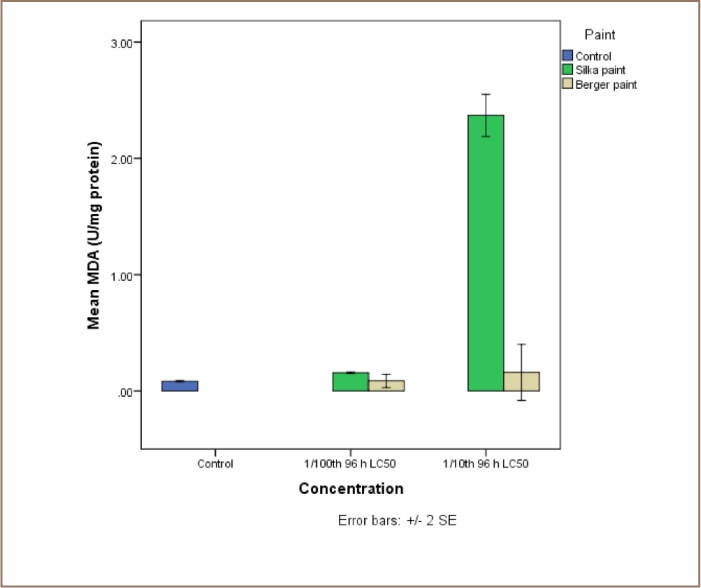
Comparative assessment of the levels of malondialdehyde (MDA) in liver tissue of catfish, Clarias gariepinus exposed to Berger and Silka antifouling paints

**Figure 5 —:**
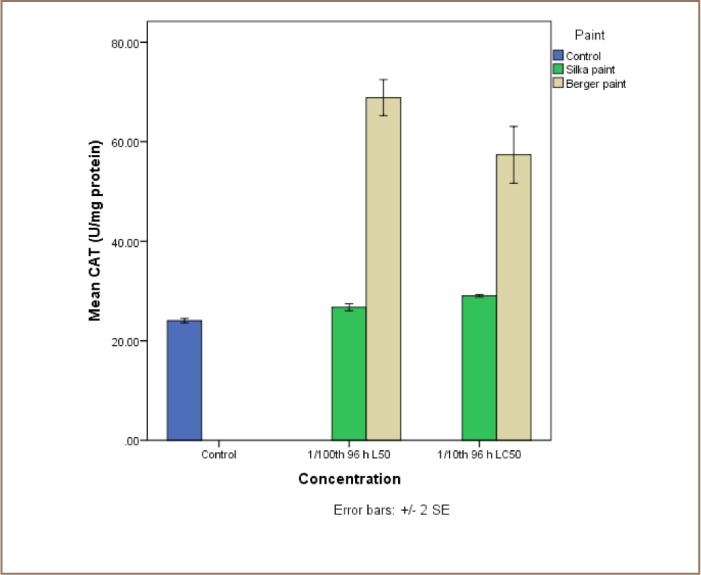
Comparative assessment of the activities of catalase (CAT) in liver tissue of catfish, Clarias gariepinus exposed to Berger and Silka antifouling paints

**Figure 6 —:**
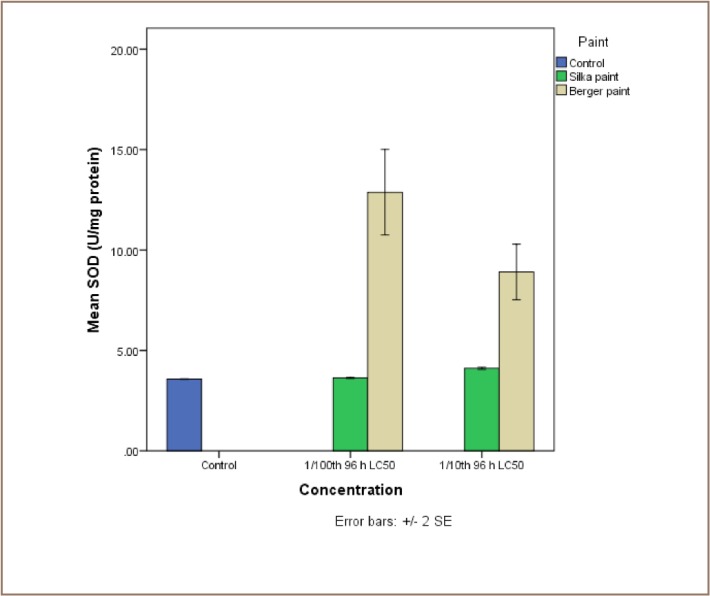
Comparative assessment of the activities of superoxide dismutase (SOD) in liver tissue of catfish, Clarias gariepinus exposed to Berger and Silka antifouling paints

**Figure 7 —:**
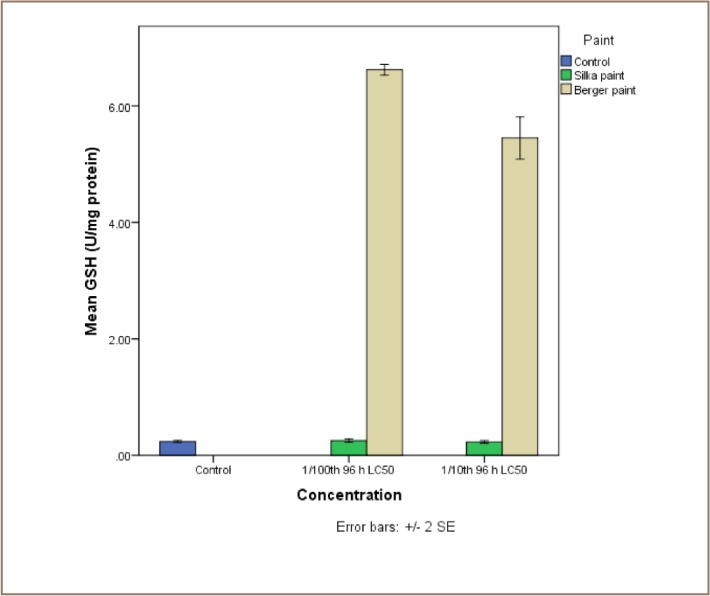
Comparative assessment of the levels of reduced glutathione (GSH) in liver tissue of catfish, Clarias gariepinus exposed to Berger and Silka antifouling paints

**Figure 8 —:**
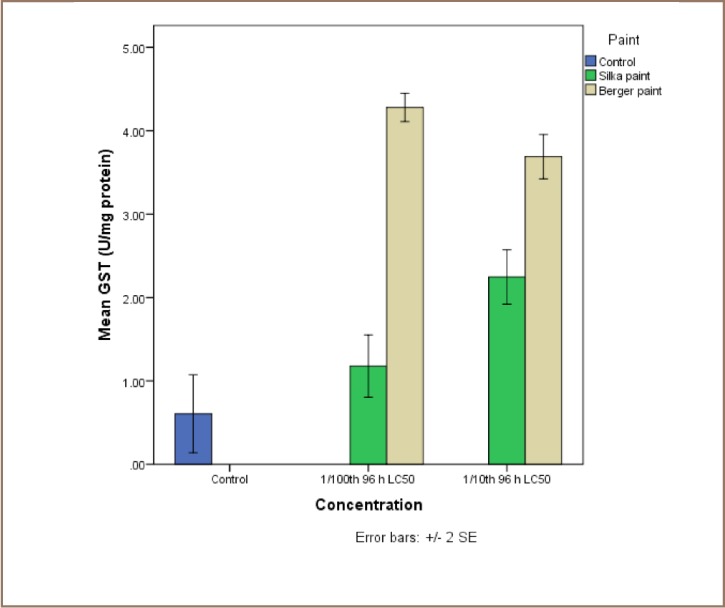
Comparative assessment of the activities of glutathione–S-transferases (GST) in liver tissue of catfish, Clarias gariepinus exposed to Berger and Silka antifouling paints

The results of the exposure of juvenile catfish to both antifouling paints are presented in Figures 4–8. Although Silka paint-exposed catfish recorded higher lipid peroxidation products, the reverse was the case for the enzyme activities, because they were all higher in the Berger paint exposed group.

The lipid peroxidation product (MDA) levels were significantly higher in catfish exposed to Silka antifouling paint than in control individuals at 1/10th and 1/100th 96-hour LC_50_ concentrations. The levels of MDA in fish exposed to 1/10th 96-hour LC_50_ values of Silka and Berger paints were 2.37±0.13 U/mg protein and 0.09±0.03 U/mg protein, respectively (*Figure 4)*. There was no significant difference between CAT activities in the control (27.39±4.25 U/mg protein) and individuals exposed to both sub-lethal concentrations of Silka paint (29.03±0.15 and 26.74±0.42 U/mg protein for 1/10th and 1/100th 96-hour, LC_50_ respectively). However, those exposed to Berger paint (68.89±2.05 and 57.39±4.02 U/mg protein) showed significant differences from both the control and Silka paint-exposed group (*Figure 5*).

The SOD activities in the liver samples of the control catfish (3.57 ±0.01 U/mg protein) were not significantly different from those in Silka paint exposed groups at 1/10th and 1/100th 96 hour LC_50_ concentrations (4.11±0.04 and 3.63±0.02 U/mg protein). However, SOD activities in those exposed to Berger paints 1/10th and 1/100th 96-hour LC_50_ concentrations (8.91±0.98 and 12.88±1.51 U/mg protein) were significantly higher than both the control and Silka exposed group. Overall, there was no significant dose dependent trend for SOD activities in the juvenile catfish exposed to both paints (*Figure 6*).

Reduced GSH levels in the catfish liver samples showed no significant differences between controls (0.24±0.01 U/mg protein) and both concentrations of Silka paint exposure (0.23±0.02 and 0.25±0.02 U/mg protein). However, levels in Berger paint-exposed fish (5.45±0.22 and 6.62±0.06 U/mg protein) remained significantly higher than both concentrations of Silka paint exposure. There was no dose dependent trend in term of levels of GSH in either treatments of either antifouling paint (*Figure 7*).

With respect to the catfish liver activities of glutathione-s-transferases, levels in the control (0.31±0.31 U/mg protein) were significantly lower than those in both Silka (2.25±0.20 and 1.17±0.23 U/mg protein) and Berger (3.69±0.19 and 4.28±0.12 U/mg protein) paints. No dose dependent relationship could be inferred for either paint (*Figure 8*).

## Discussion

The findings of the present study indicated that two common antifouling paints being sold in the Lagos markets have effects on non-target species such as catfish, potentially contributing to human food shortages. Berger antifouling paint was found to be highly toxic to the non-target catfish, C. gariepinus, in line with the Group of Experts on Scientific Aspects of Marine Environmental Protection (GESAMP)^26^ ranking of acute toxicity responses with an LC_50_ value of 0.710 mg/L. Based on the GESAMP ranking, a compound is said to be highly toxic if its LC_50_ ranges from >0.1 to ≤1 mg/L. In addition, although Silka paints were less toxic, the LC_50_ value of 11.49 mg/L falls within the slightly toxic category (i.e>10 to ≤ 100 mg/L). Given that the use of these paints is common on vessels, boats and fixed structures, often by the coastline, this raises important ecological concerns as most fish tend to spawn around these coastal areas. This adds to the pressure of other anthropogenic activities in the lagoon coastline which have been reported to be possible causal factors to fish species declines recorded in previous studies.^27^ The Lagos Lagoon is a traditional fishing ground for many riverside/coastal communities who rely on it for both subsistence and commercial purposes. The lagoon is a center for fishing, boat transportation and sand mining for the local coastal communities. Continued decline in fish populations would disrupt these already vulnerable communities.

The potential hazard of both paints in the present study is also evident in the biochemical responses elicited by the exposed catfish. The significantly higher levels of the lipid peroxidation product MDA in the Silka paint-exposed catfish points to its oxidative stress potential. Free radicals which might leach out from the paints may interact with the phospholipid bi-layer of cell membranes, resulting in damaging effects on the cells. MDA is one of the aldehydes produced when cell membranes are damaged by free radicals or reactive oxygen species.^28^ Lipid peroxidation is one of the primary events associated with cellular injury,^29^ which expresses itself in the form of tissue lesions due to DNA base oxidation.^31^

In addition to enzyme inhibition, increased enzyme activities are indicative of the onset of activities to mitigate ROS and free radicals so as to restore the redox balance in cells. When the anti-oxidative stress enzymes become overwhelmed, ROS takes over the cell system, resulting in enzyme inhibition and oxidative damage to cellular macro molecules,^30^ which could lead to lesions and mutations.^31^ The activities of enzymes associated with cellular antioxidative stress mechanisms such as CAT, SOD, GSH and GST were elevated significantly compared to the control in the catfish exposed to Berger antifouling paints. One possible explanation is that the cells of the catfish came under ROS attack and are in an active state of mitigation in other to minimize harm to the fish. While SOD acts directly on the ROS, CAT further neutralizes the attack by converting hydrogen peroxide by-products of SOD activities into water.^29^ Hydrogen peroxide radicals are cytotoxic and can be involved in the production of further active oxygen species such as singlet oxygen^29^ and thus must be quickly broken down. Thus, the similarly recorded increased activities of both enzymes in the Berger paints-exposed catfish are consistent with their mechanism of action.

There was also evidence of significantly increased GSH levels and GST activities in the liver samples of the Berger paint-exposed catfish relative to controls. Glutathione is considered to be one of the most important cellular protection mechanisms. It may act by conjugation which is catalyzed by GST, among other methods.^29^ This may explain why increased levels of the latter and the former were reported in the catfish liver samples.

One of the major effects of oxidative stress is damage to chromosomes which can be assessed using micronucleus and nuclear abnormality assays in red blood cells. The use of micronucleus induction in pollution effect monitoring is routine^31^ and has been employed in assessing the health of mudskippers (Periothalmus papilio) residing in the coastal Lagos Lagoon in a previous study.^32^ The findings from the present study indicated that both antifouling paints are potential mutagens given that there were higher numbers of cells with micronucleus and binucleus among the exposed catfish compared to controls. Micronuclei are aberrant nuclei produced during impaired mitosis and their occurrence is a simple diagnostic tool to determine the clastogenicity/mutagenicity of substances. The inductions were dose dependent, indicating that action needs to be taken to manage the entry of these paints into the Lagos Lagoon in order to prevent large-scale harm to fish. The observed clumping of red blood cells is notable and might be associated with ischemia, which could have resulted from damage to the hepatocytes.^29^ There was evidence of widespread red blood cells lysis during the assessment, and this could imply that the paints are able to cause hemolysis, thus impairing oxygen transport and other functions of red blood cells. Both paints investigated in this study are composed primarily of heavy metals such as Cu, Pb and Zn and heavy metals have been associated with the induction of cytotoxic and genotoxic effects in fish.^33^ The higher toxicity reported for Berger paint may be due to the presence of a mixture of Cu, Pb and Zn compared to Silka paint, which contains Pb only. Copper has been reported to be a potential source of ROS capable of eliciting oxidative stress,^34^ and has been linked with effects on non-target organisms and the environment.^35^ In addition, the difference in toxicity might also be contributed by other non-metallic components of the paint.

The observed tissue damage in the gills and liver might also be linked to the impact of oxidative stress in addition to physical damage caused by the paints as they solidify. Exposure-related effects in gills have been reported by Authman et al.^36^ When stress and toxicants interact with cellular components, tissue lesions and other forms of damage may be inevitable.^32^ The apparent damage to fish gills is indicative of the ability of these antifouling paints to distort the life functions of non-target species by causing damage to respiratory surfaces, thereby limiting respiratory efficiency. Observed alterations in the gill anatomy such as curved lamellae may be the effect of the exposures or a protective response of the gill to minimize the impacts of exposure.^37^

The widespread steatosis and portal inflammation observed in the liver demonstrates the damaging effects of exposure to both antifouling paints on non-target fish. Although reversible, accumulation of fat is a common cellular response to toxic compounds. It may result from inhibition of lipid excretion, increased synthesis or uptake of lipids and/or decreased lipid metabolism.^29^ However, while it lasts, it has the capacity to impair liver function due to reduced hepatocyte surface area. Stress impairs the life function and health of fish, with the potential to cause mortality or enhance vulnerability to disease.^38^ Pollution stress may reduce immunity and enhance susceptibility to parasitic infections.^39^ The disease condition of fish is also of concern to public health with respect to consumers of such food. Bamidele et al. reported the presence of Ascaris sp. in the Frillfin Gobby, Bathygobius soporator, a commonly consumed fish from the Lagos Lagoon.^37^ Given that humans can equally be infected with parasites of this species, this raises concerns regarding the potential of zoonotic transmission diseases from fish to humans. Impaired fish health could eventually result in reduced energy investment in reproduction and an overall decline in the fish catch. This would contribute to food shortages.

## Conclusions

The findings in the present study clearly demonstrate the threat posed by continued use of antifouling paints on fish in the Lagos Lagoon. These findings have opened up a debate in the Nigerian environmental circuit and local standards are needed for antifouling paints in addition to the signing of International Maritime Organization treaties. The effects on the non-target catfish species is of concern, not just to regulators, but also to the residents of the Lagos suburbs, who depend on the lagoon for fish for a significant proportion of their dietary protein. Pollution induced stress may ultimately threaten this staple food source in a developing country already concerned with food security. Furthermore, more comprehensive in situ assessments are needed in order to determine the current health status of fish populations in the Lagos Lagoon, as they continue to be exposed to potential biocides.
